# Design of a Bio-Inspired Anti-Erosion Structure for a Water Hydraulic Valve Core: An Experimental Study

**DOI:** 10.3390/biomimetics4030063

**Published:** 2019-09-06

**Authors:** Haihang Wang, He Xu, Yonghui Zhang, Siqing Chen, Zitong Zhao, Junlong Chen

**Affiliations:** College of Mechanical and Electrical Engineering, Harbin Engineering University, Harbin 150001, China; wanghaihang@hrbeu.edu.cn (H.W.); zhang.yonghui@hrbeu.edu.cn (Y.Z.); heu_chensiqing@163.com (S.C.); zhaozitong@hrbeu.edu.cn (Z.Z.); chen_junlong@hrbeu.edu.cn (J.C.)

**Keywords:** bio-inspired design, water hydraulic valve, multi-source coupling bionic, fractal structure, anti-erosion, 3D printing

## Abstract

Animals and plants have numerous active protections for adapting to the complex and severe living environments, providing endless inspiration for extending the service life of materials and machines. Conch, a marine animal living near the coast and chronically suffering from the erosion of sand in water, has adapted to the condition through its anti-erosion conch shell. Romanesco broccoli, a plant whose inflorescence is self-similar in character, has a natural fractal bud’s form. Coupling the convex domes on the conch shell and the fractal structure of Romanesco broccoli, a novel valve core structure of a water hydraulic valve was designed in this paper to improve the particle erosion resistance and valve core’s service life. Three models were built to compare the effect among the normal structure, bionic structure, and multi-source coupling bionic structures, and were coined using 3D printing technology. A 3D printed water hydraulic valve was manufactured to simulate the working condition of a valve core under sand erosion in water flow, and capture the experimental videos of the two-phase flow. Furthermore, based on the water hydraulic platform and one-camera-six-mirror 3D imaging subsystem, the experiment system was established and used to compare the performance of the three different valve cores. As a result, the results showed that the coupling bionic structure could effectively improve the anti-erosion property of the valve core and protect the sealing face on the valve core from wear. This paper presents a novel way of combining advantages from both animal (function bionic) and plant (shape bionic) in one component design.

## 1. Introduction

In fluent field, erosion is the main reason for failure in important machine parts. Solid particles, such as sand, with a fluid medium move, impact a machine parts’ surface. With the fluid’s high speed, solid particles gain high kinetic energy that damages equipment [1,2,3]. Therefore, researchers have become increasingly interested in how to resist erosion so to protect materials, reduce energy consumption, and increase economic benefit. To resist the erosion of components, using materials with high strength, stiffness, and toughness to replace standard material is common practice [4,5]. However, such practices are not economical. Another alternative method is to add a special structure that changes the flow pattern [6,7]. Researchers control a flow pattern by these structures so as to change the situation in which contact between solid particles and surfaces occur, thereby protecting the materials [8].

In nature, animals and plants have undergone tens of millions of years of evolution so that their physical characteristics can be perfectly adapted to the environment [9]. Researchers can realize these features by studying and analyzing the mechanisms in natural situations that achieve the functions on demand [10]. In recent years, researchers have become increasingly interested in bionic structures.

Researchers have shown that adding a non-smooth surface can reduce erosion with solid particles. Kazemi et al. focused on a coast that was protected from solid erosion by mangroves roots with complicated hydrodynamics [11], finding the fluid parameters, such as velocity, vorticity, shear stresses, and turbulence intensity decreased with the porosity of mangroves roots. Yin et al. [12] presented that, due to the grooves of tamarisk surface, tamarisk was protected from sand and wind erosion. A convex pattern and groove were designed and compared with that of a smooth surface to test the property of gas–solid erosion in these studies [6,7,13,14]. Qian et al. proved that a convex pattern increased the anti-erosion property and the maximum erosion rate was reduced due to the more dispersed distribution of sand particles around the pressure side and suction side, which caused the number of solid particles impacting the surface to be reduced [15]. Zhang et al. designed a bionic fan blade with grooves based on a desert scorpion and found that the fluid formed a vortex in the grooves [16]. In the research of Chen et al. and Zhang et al., the microstructure and microstrain of bionic samples after being eroded were observed by XRD and SEM, and were compared to a smooth sample [17,18]. These studies suggest that the bionic structure enhanced rotational flow in the fluid field, causing a decrease in the number and momentum of particles hitting the surface. Therefore, the corrosion damage of the bionic surface was reduced.

However, each of these previous studies adopted only a single biological feature and could not obtain perfect erosion resistance [19,20,21,22]. Artificiality cannot be designed and manufactured with the same precision and adaptability as animals and plants [23,24,25]. These features were not repaired automatically once they had been destroyed [26]. Thus, many studies pay attention to coupling bionic [25,27]. Zhang et al. presented a new approach that adopted coupling bionics to resist erosion. The desert scorpion was researched for the microstructure’s mechanism, for which which a new bionic surface was designed that combined the grooves and convex pattern. After the experiment, this structure proved to be more resistant to erosion than a single bionic structure [28]. Huang et al. focused on the tissue structure of a desert lizard’s dorsal skin (*Laudakin stoliczkana*) which combined the hard structure and groove structure [29]. On this basis, Zhang et al. simulated single particle impact on a bionic and standard surface [30]. It confirms that the bionic surface could reduce erosion by absorbing the kinetic energy of particles.

In the field of fluid mechanics, experimental techniques for the velocity measurement of flow play a crucial role [31]. Particle image velocimetry (PIV), a non-intrusive technique, continues to develop year-on-year with the development of high-speed optical capture equipment. The water hydraulic components are normally made by metals, whose body is opaque. To visually investigate the flow process inside, transparent bodies have tried to be used to capture the image information inside the pressure vessel [32,33,34]. Banaś et al. built a plastic throttle valve made by poly to track moving air bubbles in two dimensions with the help of the PIV technique [33]. The valve port field in this study had an opaque valve core, which results in a portion of the fluid domain to be blocked. This experiment process is essentially three dimensional, because one or two-dimensional techniques such as hot-wire anemometry and planar particle image velocimetry (PIV) cannot fully capture it [35]. Then, a three dimensional imaging technology [36] combined with the PIV technique was applied and its experimental platform was built in this paper.

The purpose of this paper is to adopt three biological features to make up for this shortcoming and obtain a better anti-erosion structure. Moreover, through computational and experimental research, the bionic structure’s ability to alleviate solid erosion better than other bionic structures will be verified.

## 2. Multi-Source Coupling Bionic Design

The survival of living beings lies in the ability to actively adapt to changing circumstances. For animals or plants, they may have a unique morphological structure and behavior to adapt to their harsh environments. This provides valuable inspiration for the resolution of engineering problems. However, with the complexity of engineering problems and the in-depth study of bionics, it is no longer sufficient to refer to only one biological model. This paper presents a design case of using multi-source coupling bionic based on the unit bionics. It integrates the structural and functional features of conch, romanesco broccoli, and pine cone, and designs a hydraulic valve core with anti-erosion performance.

**Figure 1:**
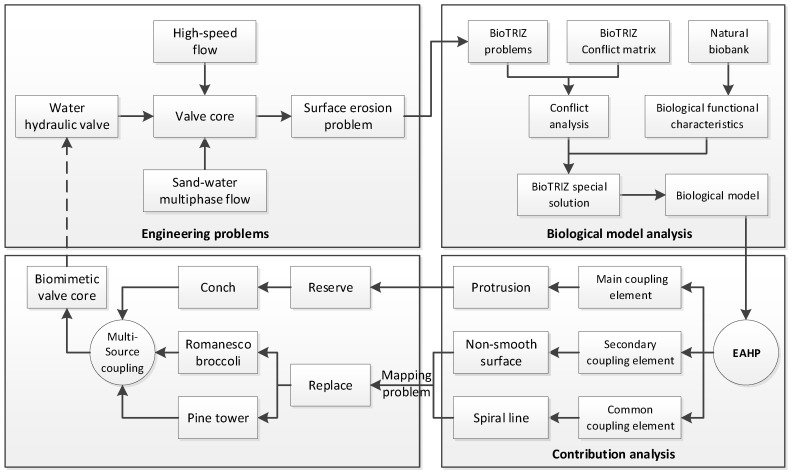
Framework of the multi-source coupling bionic.

Figure 1 presents the framework of the valve core’s biomimetic design. The erosion of the high-speed sand-water multiphase flow will cause a surface erosion problem for the water hydraulic valve core. To improve the erosion resistance of the valve core, the BioTRIZ method was used to address engineering contradictions and guide the search for biomimetic models in this paper. The BioTRIZ special solution was obtained by comprehensive biological functional characteristics and invention principle solutions given by conflict analysis. The preliminary biomimetic prototype was obtained by BioTRIZ and then the extension hierarchy process analytic was used to obtain the bionic prototyping coupling contribution to obtain the main coupling element. The primary coupling element was retained while the secondary coupling element which could have a better fractal structure and the common coupling element, whose features are difficult to map, were transformed onto other organisms. As a result, the conch convex anti-erosion structure, the fractal shape of the romanesco broccoli, and the rosette spiral structure of the pine cone were integrated to the design of a novel biomimetic anti-erosion valve core.

### 2.1. Search of the Biological Model Based on BioTRIZ

Vincent et al. [37] combined TRIZ with bionics and used TRIZ theory to make use of the advantages of historical design cases and experience, proposing the BioTRIZ theory that supports bionic design. BioTRIZ can be used to access biological strategies for solving engineering problems [38]. It can more clearly reflect the process of using biological examples to solve engineering technical contradictions.

When the hydraulic valve is under working condition, the pressure and flow at the valve port (the key field among valve seat and valve core) greatly changes. When the amount of sands in the water is large, the high-speed water flow will drive the sand to impact the valve core, which causes the valve core to wear. To improve the valve core’s particle erosion resistance, the valve core structure of a water hydraulic valve was optimally designed and bio-inspired by several natural creatures.

BioTRIZ conflict matrix is different from Altshuller’s TRIZ conflict matrix. From the perspective of biological function realization, the BioTRIZ theory reorganizes and condenses the TRIZ technology conflict matrix. The BioTRIZ theory uses six operational domains as the rows and columns of the collision matrix: Substance, structure, space, time, energy, and information. It uses 40 principles of invention as the values of the matrix. In engineering problems, reducing the grinding will improve energy efficiency and the complicity of the structure. Thus, improving energy efficiency will deteriorate the structure. According to the conflict matrix, the corresponding invention principle, the BioTRIZ special solution can be obtained, as shown in the Table 1.

The BioTRIZ special solution was combined with the biological instance library for bionic instance search. The biological case database was combined for biological case search. The result is shown in Table 2.

It is found that the shellfish non-smooth convex shell in principle No. 3 is an ideal valve core bionic model. The macroscopic non-smooth structure can improve its anti-erosion performance. Meanwhile, considering the structural characteristics of the valve core, the shuttle shape conch was selected as the original biological model.

### 2.2. Biological Model Analysis

**Figure 2:**
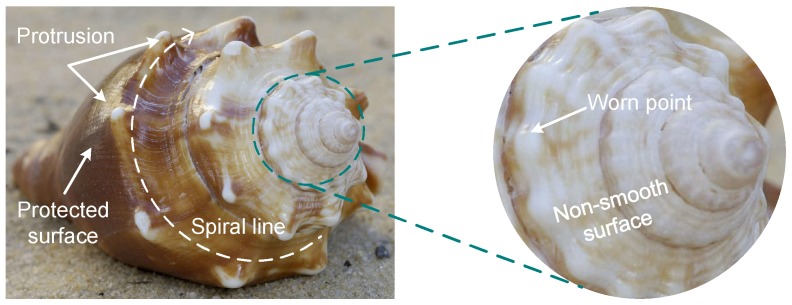
Biological characteristics of a conch.

Conchs mainly live in the shallow seabed of a coastal area and are spread all over the world. Most of populations are concentrated in the Pacific Rim and the Indian Ocean. Like other animals, mollusks such as conchs have adapted to the ever-changing environment. From the rocks washed by the sea to the deep, muddy bottom of the sea, all forms of habitat have their own special mollusk. At the sea beach and river bank where the sand is mixed in water, a large number of conchs can often be found. Conch activity is slow and often adsorbs on the reef. As shown in Figure 2, the conch is generally fusiform and the middle shoulder is raised. The overall screw shell presents a non-smooth surface. A spiral emits from the tail and extends all the way to the shoulder. There are prominent protrusions on the spiral line. Several turns of ribs and small nodules are among the spirals that give the conical outer shell a non-smooth surface. Comprehensive analysis shows that these protrusions, spirals, and non-smooth surface morphology can reduce the erosion of sediment. The outer shell of the conch is well protected against abrasion and erosion.

### 2.3. Coupling Element Contribution Analysis

Extension analytic hierarchy process (EAHP) [39] is based on the theory and method of extension set. EAHP studies the method of constructing a judgment matrix and evaluating it when the relative importance is uncertain. In this paper, the extension analytic hierarchy process is used for the coupling contribution analysis. The specific analysis steps are as follows.

The expert compares all the *N* coupling elements to give the judgment value and uses the extension interval number to quantitatively express their relative importance, thus constructing an extension interval number judgment matrix $M^{\prime }$.

(1) $$M^{\prime }={\left[m_{ij}\right]}_{N\times N}$$

$M^{\prime }$ is the standard judgment matrix, which is given by an expert. The element $m_{ij}=\left(m_{ij}^{-},m_{ij}^{+}\right)$ is an interval. $m_{ij}^{-}$ is the lower limit of the interval and $m_{ij}^{+}$ is the upper limit of the interval. *i* and *j* represent different influencing factors. In order to quantify it, the median of interval numbers $(m_{ij}^{-},m_{ij}^{+})/2$ are represented by the integers 1 to 9. 1 indicates that the two factors are equally important. 9 means one is more extremely important than the other. $m_{ij}^{t}=\left(m_{ij}^{t-},m_{ij}^{t+}\right)$ (i,j=1, 2, ⋯, N; t=1, 2, ⋯, T), given to calculate the comprehensive matrix, is the judgment interval for the $t^{\mathrm{th}}$ expert. $m_{ij}^{t-}$ is the lower limit of the interval and $m_{ij}^{t+}$ is the upper limit. The number of comprehensive extensions given by *T* experts is obtained as follows:

(2) $$M_{ij}=\frac{1}{T}⨂\left(m_{ij}^{1}+m_{ij}^{2}+\cdots +m_{ij}^{T}\right)$$

Thus, a comprehensive extension judgment matrix *M*, which combines the judgments of several experts, can be obtained.

According to the comprehensive judgment matrix *M*, find the weight vector that satisfies the consistency, which are:

1. Solve the feature vectors $x^{-}$, $x^{+}$, which are respectively the normalized eigenvector with a positive component of the largest eigenvalues of the square matrixes $M^{-}$ and $M^{+}$;
2. According to the value of $M^{-}$, $M^{+}$,
  (3) $$k=\sqrt{\sum_{j=1}^{N}\frac{1}{\sum_{i=1}^{N}m_{ij}^{+}}}$$
  (4) $$m=\sqrt{\sum_{j=1}^{N}\frac{1}{\sum_{i=1}^{N}m_{ij}^{-}}}$$
3. Calculate weight vector $S^{b}={\left(S_{1}^{b},\;S_{2}^{b},\;\cdots ,\;S_{N}^{b}\right)}^{T}=\left(kx^{-},\;mx^{+}\right)$.

According to theorem 2 presented by Gao et al. [39], the ordering weight vector *p* of each coupling element to the coupling target was calculated and the coupling analysis of the initial bio-model conch anti-erosion multi-coupling was performed. Before calculating the vector *p*, we need to sort weight vector $S^{b}$. Define $V(S_{i}^{b}\geq S_{j}^{b})$ to be the probability that $S_{i}^{b}$ is greater than $S_{j}^{b}$. The algorithm is as follows:

(5) $$V\left(S_{i}^{b}\geq S_{j}^{b}\right)=\frac{2(m_{i}x_{i}^{+}-k_{j}x_{j}^{-})}{\left(m_{j}x_{j}^{+}-k_{j}x_{j}^{-}\right)+\left(m_{i}x_{i}^{+}-k_{i}x_{i}^{-}\right)}$$

If $V(S_{i}^{b}\geq S_{j}^{b})$ is not negative, then $P^{j}=1$, $P^{i}=V\left(S_{i}^{b}\geq S_{j}^{b}\right)$. $P_{i}$ is to get the ordering weight vector *p*.

The coupling elements are respectively a clad-like protrusion, a non-smooth surface morphology, and a radioactive spiral. The experts analyzed the non-smooth surface shape and the radioactive spiral coupling element that affects the anti-erosion function and obtains the extension interval number judgment matrix $M^{\prime }$ of the function of the coupling layer. Then the extension judgment matrix is:

$$M=\left[\begin{array}{ccc} \left(1.00,1.00\right) & \left(2.78,3.40\right) & \left(3.85,4.63\right)\\ \left(0.30,0.37\right) & \left(1.00,1.00\right) & \left(1.60,2.01\right)\\ \left(0.22,0.27\right) & \left(0.51,0.65\right) & \left(1.00,1.00\right) \end{array}\right];$$

$$M^{-}=\left[\begin{array}{ccc} 1.00 & 2.78 & 3.85\\ 0.30 & 1.00 & 1.60\\ 0.22 & 0.51 & 1.00 \end{array}\right];\;M^{+}=\left[\begin{array}{ccc} 1.00 & 3.40 & 4.63\\ 0.37 & 1.00 & 2.01\\ 0.27 & 0.65 & 1.00 \end{array}\right];$$

$$x^{-}={\left(0.635\;0.226\;0.139\right)}^{T};\;x^{+}={\left(0.631\;0.228\;0.141\right)}^{T}$$

where,

k=0.969,m=1.023

Then,

$$S_{1}^{b}=\left(0.62\;0.65\right),\;S_{2}^{b}=\left(0.22\;0.23\right),\;S_{3}^{b}=\left(0.13\;0.14\right)$$

$$P_{1}^{b}=V\left(S_{1}\geq S_{3}\right)=34.13,\;P_{2}^{b}=V\left(S_{2}\geq S_{3}\right)=13.34,\;P_{3}^{b}=1$$

Normalization gives the weight vector of each coupling element to the functional target as $p={\left(0.704\;0.275\;0.021\right)}^{T}$.

It can be seen that the convex hull structure, non-smooth surface morphology and spiral curve are the coupling elements, and the contribution of the 3 coupling elements are respectively ${\left(0.704\;0.275\;0.021\right)}^{T}$. Therefore, the convex hull structure is the main coupling element, the non-smooth surface morphological coupling element is the secondary main coupling element, and the spiral curve is a common coupling element.

### 2.4. Multi-Source Coupling Biomimetic Design

Through the above methods, we found a primary model for solving engineering problems: Conch. However, when constructing the core, the non-smooth surface morphology and spiral curve of the conch are difficult to map to the valve core. However, these two coupling elements are not the main coupling elements and can be simplified in a certain way. Thus it is necessary to continue to search for new bio-models to replace the non-smooth surfaces and spiral curves of a conch.

#### 2.4.1. Replacement of the Common Coupling Element

When mapping the conch to the valve core, it was found that the spiral line of the protrusions greatly decrease the amount of protrusions and result in unnecessary structural complexity. Therefore, it is necessary to find another biometric model to replace the spiral line of the conch.

**Figure 3:**
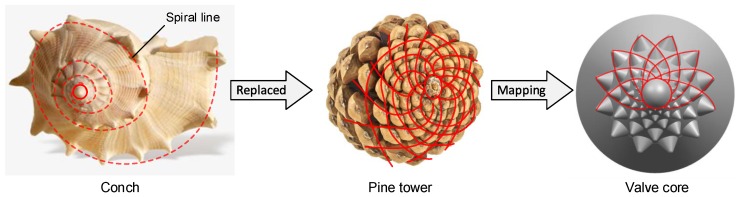
Biomimetic mapping of a pine tower.

Pine tower, the fruit of the pine tree, has brown, multi-layered scales with pine nuts sandwiched between the scales. The scales of fresh pine towers have small thorns. When mature, the skin is lignified and forms a hard-outer shell. As shown in Figure 3, the arrangement of the scales on the pine tower is a rosette type, and the scales of each layer are mutually fitted and the structure is stable. The pine tower has a simple spiral structure and can arrange more pine nuts in a limited space. Therefore, the Fibonacci spiral arrangement of the pine cone can simplify the modeling process, densify the main coupling element, and strengthen the main coupling element strength.

#### 2.4.2. Replacement of the Secondary Coupling Element

The fractal geometry theory proposed by Benoit B. Mandelbrot [40] presented a branch of mathematics that explores the complex forms of nature. There are many complex objects in nature, such as coastlines, mountain contours, floating clouds, and stars. It is difficult to describe them using traditional geometric methods and can only be approximated by regular shapes. Fractal geometry is a geometry with irregular geometry as the object of study. It can deal with the appearance of non-smooth and irregular self-similarity and feature length without appearance in nonlinear systems. It can also more deeply describe the natural forms which may be disorganized in nature [41]. The fractals of nature are divided into regular fractals and random fractals. Regular fractals are carefully constructed by mathematicians, which strictly satisfy self-similarity such as the Koch snowflake curve [42] and the Shelbinski sierpinski rug curve [43]. The vast majority of the rest are random fractals. They are extremely irregular and extremely matte, but they are self-similar in appearance.

**Figure 4:**
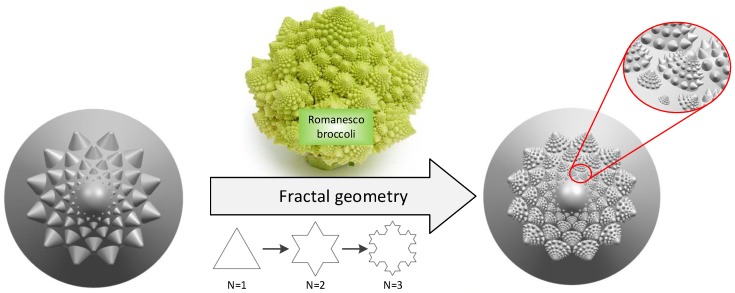
Biomimetic mapping of romanesco broccoli using fractal theory.

When using a fractal model to characterize an object, a random process (or recursive model) was constructed and iterated step by step until the richness of the generated texture details are met. Therefore, a regular fractal structure is used to replace the non-smooth surface morphology of the surface of the conch. There is a broccoli growing in the northern part of the Mediterranean Sea, called a romanesco broccoli. Its shape resembles a pagoda and has a fractal structure, as shown in Figure 4. Each romanesco broccoli is composed of the same tower-shaped pagoda and each cluster of romanesco broccoli is made up of smaller shapes. Each cluster of romanesco broccoli is arranged according to the Fibonacci spiral.

The tower-like structure of this romanesco broccoli is similar to the telescopic protrusion of the conch and its multi-level self-similarity can approximate the non-smooth surface texture of the conch shell. The fractal structure also greatly increases the spatial density of the main coupling element and can further enhance the ability to resist erosion. As shown in Figure 4, a multi-source coupling bionic valve core was designed combined with the anti-erosion function of the conch, the spiral curve of the pine tower, and the fractal shape of the romanesco broccoli.

## 3. Bionic Design of the Valve

### 3.1. Structure Design of Valve Core

According to the model for describing the fractal characters in [44], a unique property of a fractal object is its measure M(l) and the measured dimension l that obey the following relationship:

(6) $$M\left(l\right)∼l^{D}$$

In the above formula, *D* is the fractal dimension and M(l) can be the mass, volume, area, or length of an object. Another property of a fractal object is their cumulative number and the size distribution of objects obeying the following relationship [45].

(7) $$N\left(l\geq \lambda \right)={\left(\frac{\lambda_{max}}{\lambda }\right)}^{D}$$

In Equation (7), λ is the diameter of object and *N* is the number of pore diameters equal to or greater than λ. $\lambda_{max}$ is the largest diameter of the object and *D* is the fractal dimension.

The specific structural parameters of the fractal were designed according to the formula. There is a certain functional relationship within the regular fractal, which limits the freedom of design. When the valve was working, the valve core had to match with the valve seat, so the maximum diameter of the valve core remained smaller than the minimum inner diameter of the valve seat. Due to manufacturing accuracy, the minimum feature was greater than the machining accuracy. The diameter of the bottom surface of the valve core was 12.5 mm and the minimum inner diameter of the valve seat was 16 mm. Furthermore, these features cannot interfere with each other. Through the calculation and analysis and CAD mapping measurement, a set of optimization parameters for solving contradictory problems was obtained.

**Figure 5:**
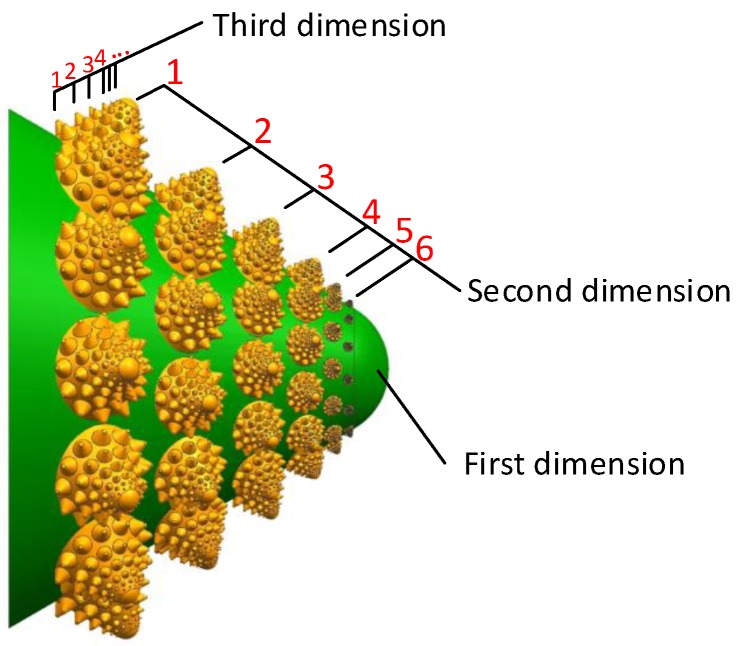
Bionic fractal structure at the front of the valve core.

The characteristics of the valve core are generally conch convex-packed structures, which are shaped to have different sizes and the overall performance is a non-smooth surface structure. The convex hulls are arranged in the rosette arrangement of the pine tower to form a certain spiral structure. There are three dimensions in the fractal structure. Each dimension has a total of six convex hulls and each layer is evenly distributed around the axis of the 12 convex hulls. In order to simulate the rosette arrangement of the pine cone and then decrease the damage degree by changing the flow direction, the convex hulls of the adjacent layers are different by 30° around the central axis. The convex hull is a cone with a cone apex angle of 60°. The first dimension valve core has a diameter of 12.5 mm. As shown in Figure 5, the green cone is the first dimension. The yellow cones are the second dimension. The smaller cones on the yellow cones are the third dimension. The layer numbers of the second and third dimension are noted in Figure 5. The different layers of the same dimension differ from the first layer by a factor of 0.2. In addition, according to the rules of the first five layers, the sixth layer should be zero. To simulate the structure of the organism, there is no mutation to zero. Thus, the relatively unimportant sixth layer was set to 0.1 times than the first layer. Then, the diameter minifications are defined as 0.8, 0.6, 0.4, 0.2, and 0.1, respectively.

The specific parameters are given in Table 3. There are small cones on each layer of the second dimension. Since the two-dimensional table cannot express the three-dimensional information, the parameters of the small cones of each layer are stacked in the table of the third row.

**Figure 6:**
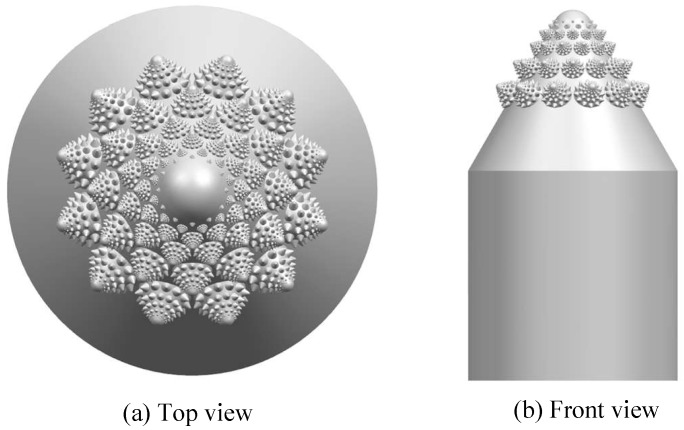
Biomimetic valve core.

Using the multi-source coupling bionics idea, the conch convex hull structure, the romanesco broccoli fractal structure, and the pine cone spiral are integrated into the valve core. As a result, CAD modeling was built using Siemens NX 10.0 software (Siemens Product Lifecycle Management Software Inc., Plano, TX, USA), as shown in Figure 6.

### 3.2. Simplified and Comparative Design of Bionic Valve Core

**Figure 7:**
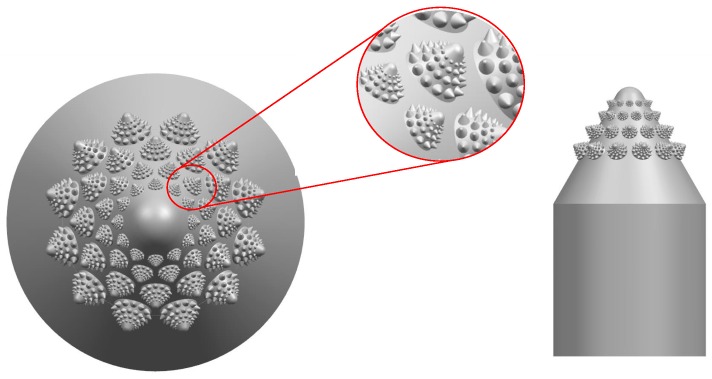
Simplified bionic valve core (model C).

As shown in Figure 6 and Table 3, the fifth and sixth layers of the second-dimensional convex hull have a maximum diameter of 0.5 mm. The third-dimensional convex hull on the second is 0.01 mm, which is beyond the scope of human vision. The metric of roughness was reached. Ordinary 3D printing does not have a high precision structure. Due to its fine structure and arrangement on the top of the valve core, the effect on fluid flow is minimal. In order to facilitate the manufacture of the valve core, the fifth and sixth layers of the second dimension are removed, leaving only the four-layer structure as shown in Figure 7.

**Figure 8:**
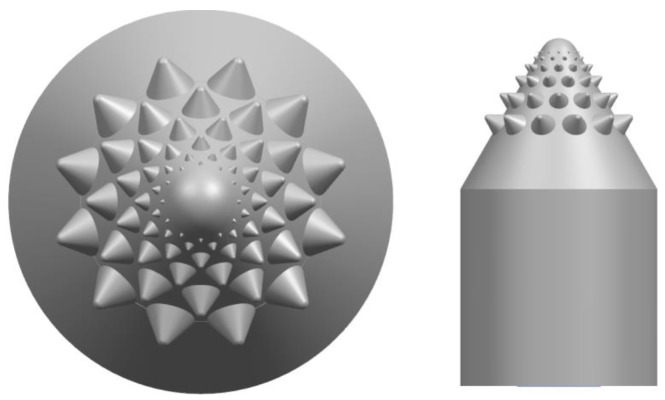
Valve core with simplified coupling element (model B).

**Figure 9:**
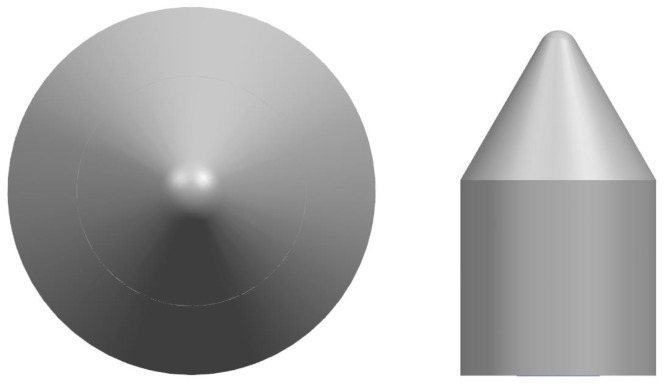
Original valve core (model A).

In order to testify the contribution of the bionic valve core main coupling element and the secondary coupling element to the erosion performance, a coupling element simplified valve core and a common valve core with only the main coupling element and the general coupling element were designed. The coupling element simplifies the valve core, that is, only the conch convex hull structure and the pine tower rosette spiral structure were combined to remove the fractal structure, as shown in Figure 8. The ordinary valve core, that is, all bionic coupling elements were removed, as shown in Figure 9.

### 3.3. Design of Bionic Valve Core and Seat

**Figure 10:**
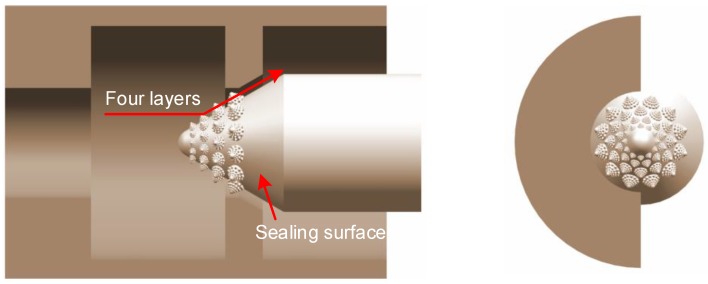
Structure of valve port field.

The bionic structure on the valve core is aimed at improving the performance of sand erosion resistance. Figure 10 is a structural section view of the valve port basin. The high-speed water stream carries high-speed moving sands and wears the front surface of the bionic valve core. The bionic valve core structure reduces the speed of the sands and changes its flow direction. The sealing surface is protected by the bionic anti-erosion structure in this paper.

### 3.4. Design of the Test Valve

**Figure 11:**
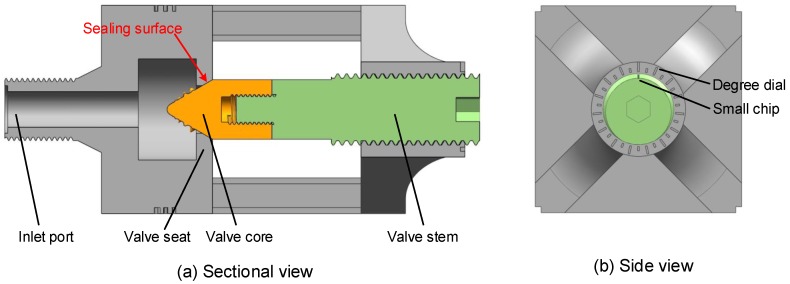
Structure of the test valve.

Figure 11 shows the structure and components of the test valve, which is guided by the bionic valve core in Section 3.3. The valve body is hollowed out on all sides to facilitate the observation of the flow state of water flowing vertically through the valve core. The observation window is 45 mm × 50 mm (Figure 11a). The bottom of the valve body is also hollowed out, which not only fills the light, but also observes the flow state in the horizontal direction of the water flow. The total height of the valve body is 155 mm and the length and width are each 70 mm.

The valve opening was designed from 0 mm to 4 mm. As shown in Figure 11b, the degree dial coordinating the small chip can change the valve opening by rotating the valve stem. The pitch of screw is 3 mm.

## 4. Experimental Results and Discussion

### 4.1. Experiment Equipment

**Figure 12:**
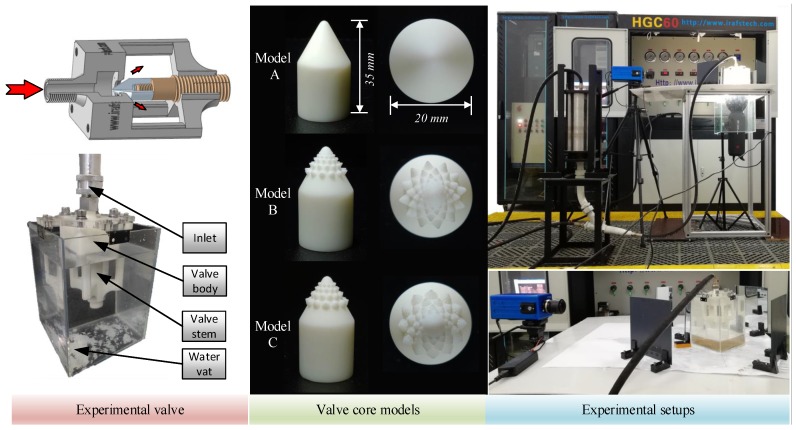
Hydraulic valve and its experimental setups.

In order to test the valve core’s effectiveness, a hydraulic test bench was established, which was divided into a water hydraulic transmission subsystem, an electrical control subsystem, and an experimental signal acquisition subsystem. The frequency inverter (Schneider Altivar 610, Paris, France) ensured suitable power for the hydraulic test bench through the adequate control of the water pumps working frequency (input pressure). The water pump used in the transmission system allowed for a constant supply of pressured water, which eliminated the shortcomings in terms of pressure fluctuations owing to subsequent improvements in AC frequency conversion technology. The hydraulic pump provided 0.2–6 MPa pressure. The hydraulic control system was controlled by the electric control subsystem, the initial parameters of the experiment were set, the experimental data acquisition subsystem was used to obtain experimental data, and the experimental results were analyzed. The experimental schematic and experimental setup are shown in Figure 12.

A high-speed camera (Revealer 5KF10, Hefei, China) with a 60 mm Nikkor lens (Tokyo, Japan) was used to get high-speed sand and water flow. The three valve core models were manufactured by using 3D printing technology. Its 3D printing accuracy was 0.025 mm, which meet the structure requirement of model C. The 3D printer used in this research is Formlabs Form 2 produced by Formlabs Inc., Somerville, MA, USA. Solid particles in the fluid were added through a mixer. Since the upper and lower pressures were uniform, the sand particles were mixed into the fluid under the force of gravity. The experimental sand-water flow conditions are listed in Table 4.

The thread on the upper part of the valve body was used to connect the water hydraulic test bench to the inlet of the valve. The narrow passage at the front end of the valve core and the large chamber greatly stabilized the flow of water into the flow before the valve core. In order to observe the flow of fluid through the valve, the valve body needed to use highly transparent plexiglass. However, under sand erosion, the transparency of the plexiglass valve body was easily destroyed, resulting in an inefficient recycling of the valve body. In order to solve this problem, a semi-hollow valve body was proposed. Putting the valve body into the water tank not only helped to carry out the anti-corrosion test, but also to observe the speed at which the erosion particles passed through the valve core.

### 4.2. Experiment Result

**Figure 13:**
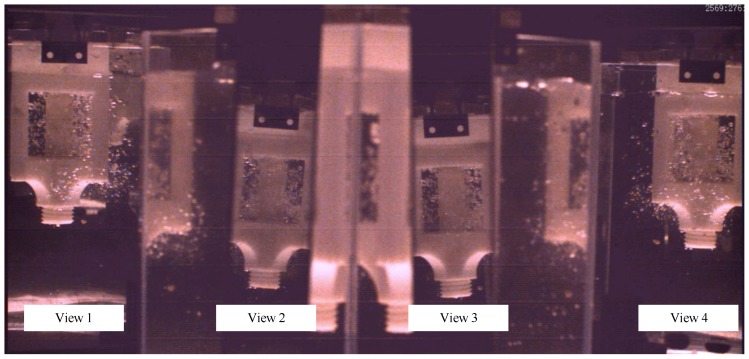
Experiment image examples from four sides.

Using the high-speed camera and one-camera-multi-mirror three-dimensional imaging method [36], the videos from the four sides of the valve were captured and saved. For example, Figure 13 shows a frame of the experiment videos. Its resolution is 1344 ∗ 628 pixels. The frame rate of the selected experiment video to be analyzed is 1794 frames per second. The exposure time was 400 μs. So, the time difference between two consecutive frames of image was only about 0.557 ms.

Through the observation of the experiment’s images, the sand mixed in water was so little in size that the resolution of the images could not provide clear information on the sand track. Meanwhile, the sealing surface could not be captured by camera due to the valve port’s structure limitation. There were plenty of air bubbles generated among the valve port field. Thus, the authors chose the velocity of the air bubbles in the water flow as a reference to the sand velocity. The erosion caused by the kinetic energy of the sands mixed in water flow. The energy of the sands hitting the sealing surface depended on sand velocity, when the other flow or particle conditions were the same. So, the measurement of the velocity vector of bubble cluster near the sealing surface could provide a credible reference for the potential erosion on the sealing surface of the valve core. The flow pattern and preformation of the valve can be discussed.

### 4.3. Result Analysis

Using PIV technology [46], the experiment’s images of the valve port field were analyzed, which mapped flow quantitatively, and got the velocity of the bubble or little sands (just a little part) fixed in water flow. The resolution of each frame of the observed area from four views were all 204 ∗ 282 pixels. In the PIV algorithm, the important settings were as follows: The correlation algorithm, fast Fourier transform (FFT) correlation with multiple passes and deforming windows, was used. The interrogation area in Pass 1 was 32 pixels, the step for image detection was 16 pixels, which is 50% of the interrogation area. The interrogation area in Pass 2 was 16 pixels, with a step of 8 pixels. In Pass 3, the interrogation area was 8 pixels with a step of 4 pixels. Due to the low resolution of the experiment images, 5 times repeated correlation function was used to enhance the data yield.

After PIV analysis, the lateral velocity *u* and vertical velocity *v* were limited in the interval between −2 px/frame to 2 px/frame. The few velocity vectors beyond this interval were considered to be inconsistent with the actual flow rate and were eliminated as errors. If *v* is less than zero, it means that the direction of this velocity vector on the longitudinal axis is upward, which means the bubbles on there are the floating ones driven by buoyant force. If *v* is more than zero, it means that the direction of this velocity vector on the longitudinal axis is downward, which means the bubbles on there are the floating ones driven by flow force.

**Figure 14:**
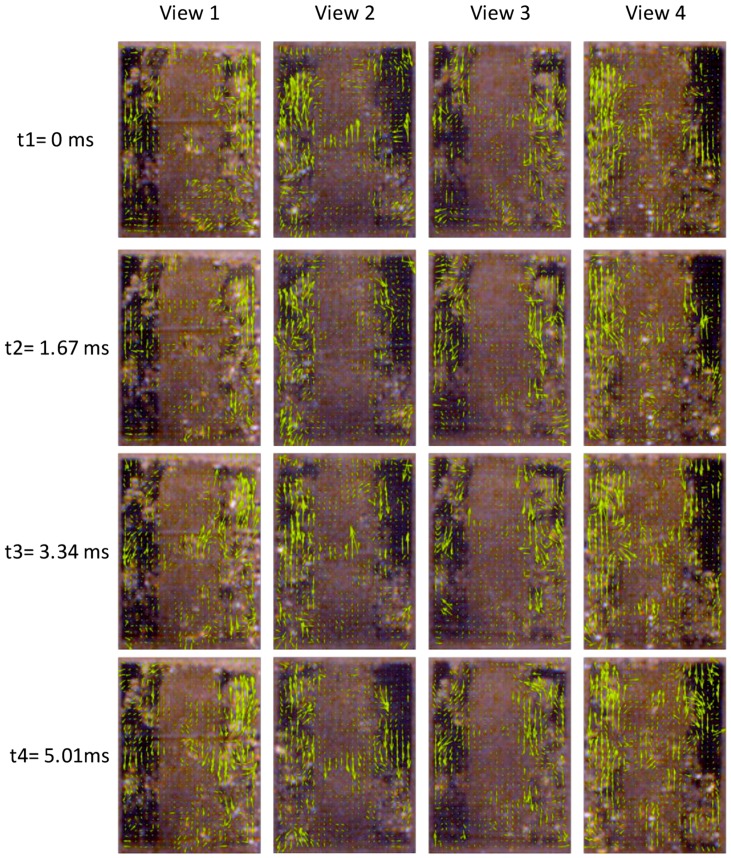
Velocity vector drawings of particle image velocimetry (PIV) results for a short period of time.

To present the PIV analysis process, four frames of image were selected as an example, whose time interval of each other is only 1.67 ms. The PIV analysis results of the selected four frames are presented in Figure 14.

The velocity of the top area bubbles was bigger than that of the lower area. It got the maximum on the valve port area, where the water mixed with sands flows out of the cabined gap between valve seat and valve core. The bubble motion direction were partly upward because of the buoyancy of water. The jetting bubbles were quite small, which maybe shelter from the outside floating bubbles.

**Figure 15:**
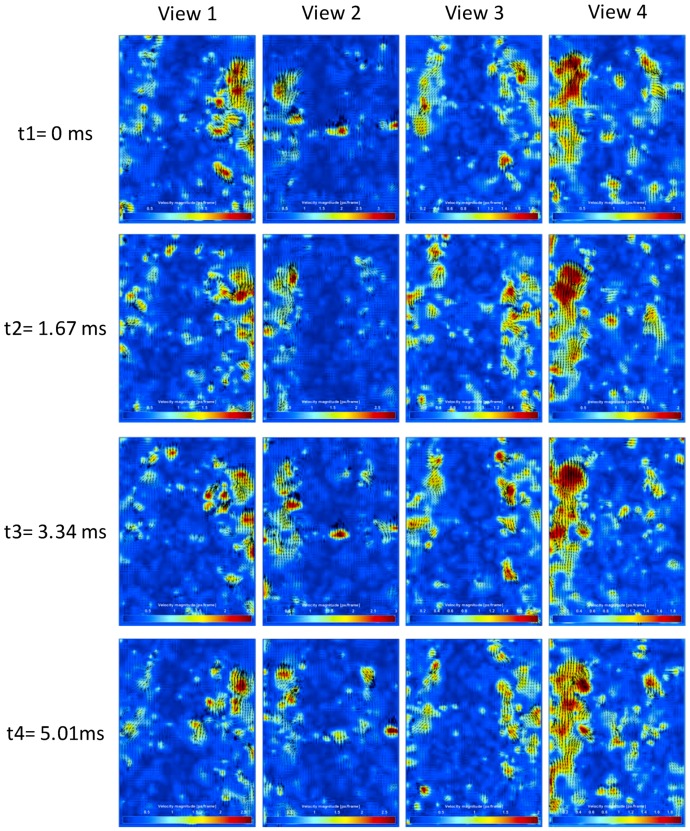
Velocity magnitude drawings of PIV results.

Figure 15 shows the velocity magnitude distribution of the PIV results in Figure 14. It clearly presents the clusters distribution of the moving bubbles and their velocity. Based on the analysis results of Figure 15, the moving bubbles among the center area of the images were rarely detected, due to the background (opaque valve core) and light interference. The bubble cluster among the valve port field were randomly distributed around the valve core. Using the three-dimensional imaging method, the bubbles missing tracking from one view could further be detected from another view. Then, the moving bubbles in the experiment images were fully tracked.

The velocity vector of $i^{\mathrm{th}}$ row $j^{\mathrm{th}}$ column from view *d* is defined as ${}^{d}V_{ij}\left(u_{ij},v_{ij}\right)$. The velocity vector matrix of a frame is defined as ${{[}^{d}V_{ij}\left(u_{ij},v_{ij}\right)]}_{m\times n}$. d=1, 2, 3, 4. i=1, 2, …, *m*. *j* = 1, 2, …, *n*. *m* is the lateral vector number of the PIV velocity results, which is equal to 50. And *n* is the vertical vector number, which is equal to 69.

(8) $$s_{i}=\sum_{d=1}^{4}\sum_{j=1}^{n}v_{ij}\;\mathrm{for}\;v_{ij}\geq 1$$

$s_{i}$ is the sum of $v_{ij}$ of the velocity vectors whose $v_{ij}\geq 1$ in a frame of image. So, the floating bubbles’ velocity will not be considered in $s_{i}$.

In order to analyze the vertical velocity distribution, 50 frames of original experimental images from four views were randomly selected from 4 ∗ 5 experiment videos. These five high-speed experiment videos were the selected fragments whose multiphase flow were clear, stable and lasted for 5 ∗ 3 s in total.

(9) $$S_{i}=\sum_{f=1}^{50}s_{i}$$

$S_{i}$ can represent the common vertical velocity on the depth of *i*. As *i* increases, the velocity of the bubbles will be smaller and smaller due to resistance of water and the buoyancy of the bubbles. There are also fewer and fewer bubbles that can reach the deeper position, which corresponds to a lower $S_{i}$.

**Figure 16:**
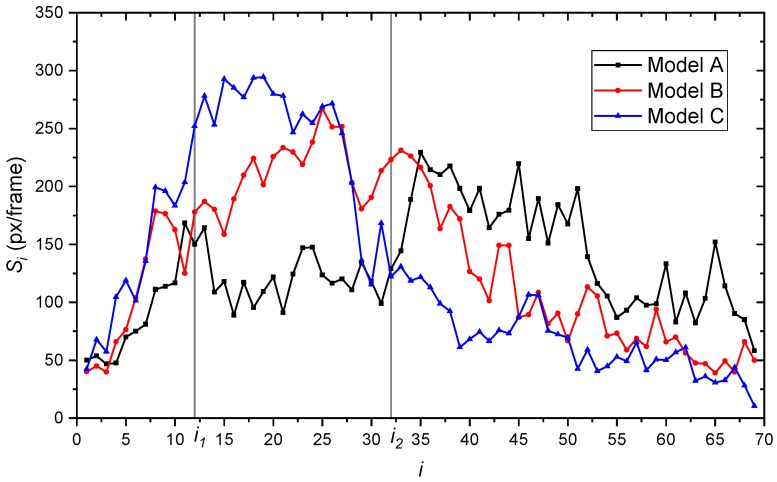
Vertical velocity statistic distribution of the three valve core models.

Figure 16 shows the $S_{i}$ changes in the *i* interval between 1 to 69. The interval can be roughly divided into three subintervals based on the changes of $S_{i}$, which are 1-$i_{1}$, $i_{1}$-$i_{2}$, and $i_{2}$-69. $i_{1}=12$. $i_{2}=32$. It is noticed that from 1 to $i_{1}$, all the $S_{i}$ of the three models greatly increase. This subinterval is near the outlet of valve port. Thus, the bubbles size and position are limited around the valve port field. The bubbles rapidly expand in size and grow into bubble cluster. After the growing interval, model C had the largest $S_{i}$ at a shallower position, compared with model A and B. Its $S_{i}$ decreased when i>27. When $i>i_{2}$, the $S_{i}$ of model C began to fall generally to less than 11 px/frame at the bottom of the experiment’s images. Compared with model A and B, the velocity condition of model C was the best. The number of fast moving bubbles at the interval from $i_{2}$ to the bottom was less than the others in common. This proves that the bionic structure on the valve core plays a positive role in reducing the flow velocity and further improve the service time of the sealing face. Compared with model C, the $S_{i}$ of model A and B had a longer distance to growing the bubbles. This may be concerned with the fractal structure of model C. The non-smooth structure on the valve core significantly influenced the flow velocity and direction. The trend on vertical velocity of model B and C was similar, with a better performance from model C. Compared with model B and C, model A with the original structure had more numbers of fast moving bubbles. This means that when the water flows out into valve and through sealing surface, the sand mixed in the water will correspondingly have more energy. Overall, the experiment results testified the effectiveness of the multi-source coupling bionic design of the valve core structure.

## 5. Conclusions

A novel valve core structure was designed using TRIZ, bionics, and fractal theory to improve the erosion resistance of a valve. The biological model was established by TRIZ and bionics, and the main factors that realized the function of the biological model were identified by the extension analytic hierarchy process. The detailed parameters of the valve core structure were determined by TRIZ and Fractal methods, and the erosion resistance of the non-smooth surface of the convex shell was testified by experiment. The combination of these three innovative methods improved the design efficiency and provided new enlightenment and innovative ideas for peer research in the bionic design of a water hydraulic valve. 

## Figures and Tables

**Table 1:** Invention principle solutions given by the BioTRIZ conflict matrix.

Engineering Field	Structure
Energy	1, 3, 5, 6, 25, 35, 36, 40

**Table 2:** Partial biological instance library corresponding to the principle of local quality in invention principles.

Number	Invention Principle	Explanation	Biological Instance
No. 3	Partial quality	A. Change the uniform structure of the object or environment into a non-uniform structure;	A. The non-smooth structure of the lotus leaf surface achieves a self-cleaning function;
		B. Make different parts of the composition object perform different functions;	B. The grooved crack of the red willow bark provides anti-sand erosion resistance;
		C. Make a part of the composition of the object to the maximum effect.	C. The grooved structure of the dolphin skin or shark skin;
			D. The non-smooth convex hull shell of shellfish.
			⋯⋯

**Table 3:** Diameter of the convex hull.

Bottom Diameter (mm)	First Layer	Second Layer	Third Layer	Fourth Layer	Fifth Layer	Sixth Layer
First dimension (mm)	12.5	—	—	—	—	—
Second dimension (mm)	2.5	2.0	1.5	1.0	0.5	0.25
Third dimension (mm)	0.5, 0.4, 0.3, ⋯	0.4, 0.32, 0.24, ⋯	0.3, 0.24, 0.18, ⋯	0.2, 0.16, 0.12, ⋯	0.1, 0.08, 0.06, ⋯	0.05, 0.04, 0.03, ⋯

**Table 4:** Erosion wear experiment conditions.

Erosion Particle	Particle Diameter (mm)	Pressure of Hydraulic Platform (MPa)	Average Mass Flow Rate (g·s^−1^)
Sand	0 to 2	0.45 ± 0.01	1.82 ± 0.5

## References

[1] Bhushan B. (2007). Adhesion of multi-level hierarchical attachment systems in gecko feet. J. Adhesion Sci. Technol..

[2] Desale G.R., Paul C.P., Gandhi B.K., Jain S.C. (2009). Erosion wear behavior of laser clad surfaces of low carbon austenitic steel. Wear.

[3] Han Z.W., Yin W., Zhang J.Q., Jiang J.L., Mu S.C., Ren L.Q. (2013). Erosion-resistant surfaces inspired by Tamarisk. J. Bionic Eng..

[4] Qu M.N., Liu S.S., He J.M., Feng J., Yao Y.L., Hou L.G., Ma X.R. (2016). Bioinspired durable superhydrophobic materials with antiwear property fabricated from quartz sands and organosilane. J. Mater. Sci..

[5] Smith G.M., Resnick M., Flynn K., Dwivedi G., Sampath S. (2016). Nature inspired, multi-functional, damage tolerant thermal spray coatings. Surf. Coat. Technol..

[6] Han Z.W., Zhang J.Q., Ge C., Jiang J.L., Ren L.Q. (2011). Gas-solid erosion on bionic configuration surface. J. Wuhan Univ. Technol.-Mater. Sci. Ed..

[7] Han Z.W., Zhang J.Q., Ge C., Lu Y., Jiang J.L., Liu Q.P., Ren L.Q. (2010). Anti-erosion function in animals and its biomimetic application. J. Bionic Eng..

[8] Nove-Josserand C., Hebrero F.C., Petit L.M., Megill W.M., Godoy-Diana R., Thiria B. (2018). Surface wave energy absorption by a partially submerged bio-inspired canopy. Bioinspir. Biomim..

[9] Bhushan B., Jung Y.C. (2011). Natural and biomimetic artificial surfaces for superhydrophobicity, self-cleaning, low adhesion, and drag reduction. Prog. Mater. Sci..

[10] Han Z.W., Feng H.L., Yin W., Niu S.C., Zhang J.Q., Chen D.B. (2014). An efficient bionic anti-erosion functional surface inspired by desert scorpion carapace. Tribol. Lubr. Technol..

[11] Kazemi A., Van de Riet K., Curet O.M. (2018). Drag coefficient and flow structure downstream of mangrove root-type models through PIV and direct force measurements. Phys. Rev. Fluids.

[12] Yin W., Han Z.W., Feng H.L., Zhang J.Q., Cao H.N., Tian Y. (2017). Gas-solid erosive wear of biomimetic pattern surface inspired from plant. Tribol. Trans..

[13] Han Z.W., Zhang J.Q., Ge C., Wen L., Ren L.Q. (2012). Erosion resistance of bionic functional surfaces inspired from desert scorpions. Langmuir.

[14] Han Z.W., Zhu B., Yang M.K., Niu S.C., Song H.L., Zhang J.Q. (2017). The effect of the micro-structures on the scorpion surface for improving the anti-erosion performance. Surf. Coat. Technol..

[15] Qian Z.D., Dong J., Guo Z.W., Wang Z.Y., Wang F. Study of a bionic anti-erosion blade in a double suction centrifugal pump. Proceedings of the Asme Fluids Engineering Division Summer Meeting.

[16] Zhang J.Q., Han Z.W., Yin W., Wang H.Y., Ge C., Jiang J.L. (2013). Numerical experiment of the solid particle erosion of bionic configuration blade of centrifugal fan. Acta Metall. Sinica-Engl. Lett..

[17] Chen G.M., Schott D.L., Lodewijks G. (2017). Bionic design methodology for wear reduction of bulk solids handling equipment. Part. Sci. Technol..

[18] Zhang J.Q., Chen W.N., Yang M.K., Chen S.Q., Zhu B., Niu S.C., Han Z.W., Wang H.Y. (2017). The ingenious structure of scorpion armor inspires sand-resistant surfaces. Tribol. Lett..

[19] Faivre J., Sudre G., Montembault A., Benayoun S., Banquy X., Delair T., David L.J.S.M. (2018). Bioinspired microstructures of chitosan hydrogel provide enhanced wear protection. Soft Matter.

[20] Sui Q., Zhou H., Zhang H.F., Feng L., Yang L., Zhang P. (2017). Effect of alternate biomimetic coupling units on dry sliding wear resistance of gray cast iron. J. Mater. Res..

[21] Tong J., Mohammad M.A., Zhang J., Ma Y., Rong B., Chen D., Menon C. (2010). DEM numerical simulation of abrasive wear characteristics of a bioinspired ridged surface. J. Bionic Eng..

[22] Zhang H.F., Zhang P., Sui Q., Zhao K., Zhou H., Ren L.Q. (2017). Influence of multiple bionic unit coupling on sliding wear of laser-processed gray cast iron. J. Mater. Eng. Perform..

[23] Yang W.S., Zhou H., Sun L., Wang C.W., Chen Z.K. (2014). Effect of biomimetic coupling units’ morphologies on rolling contact fatigue wear resistance of steel from machine tool rolling tracks. Opt. Laser Technol..

[24] Yuan Y.H., Zhang P., Zhao G.P., Gao Y., Tao L.X., Chen H., Zhang J.L., Zhou H. (2018). Effects of laser energies on wear and tensile properties of biomimetic 7075 aluminum alloy. J. Mater. Eng. Perform..

[25] Zhang Z.H., Shao F.X., Liang Y.H., Lin P.Y., Tong X., Ren L.Q. (2017). Wear behavior of medium carbon steel with biomimetic surface under starved lubricated conditions. J. Mater. Eng. Perform..

[26] Oucif C., Voyiadjis G.Z., Rabczuk T. (2018). Modeling of damage-healing and nonlinear self-healing concrete behavior: Application to coupled and uncoupled self-healing mechanisms. Theor. Appl. Fract. Mech..

[27] Zhou H., Sun N., Shan H.Y., Ma D.Y., Tong X., Ren L.Q. (2007). Bio-inspired wearable characteristic surface: Wear behavior of cast iron with biomimetic units processed by laser. Appl. Surf. Sci..

[28] Zhang J.Q., Han Z.W., Ma R.F., Yin W., Lu Y., Ren L.Q. (2013). Scorpion back inspiring sand-resistant surfaces. J. Cent. South Univ..

[29] Huang H., Zhang Y., Ren L.Q. (2012). Particle erosion resistance of bionic samples inspired from skin structure of desert lizard, laudakin stoliczkana. J. Bionic Eng..

[30] Zhang Y.H., Huang H., Ren L.Q. (2014). Erosion wear experiments and simulation analysis on bionic anti-erosion sample. Sci. China-Technol. Sci..

[31] Butcher D., Spencer A. (2019). Spurious PIV vector correction using linear stochastic estimation. Fluids.

[32] Stryczek J., Antoniak P., Jakhno O., Kostyuk D., Kryuchkov A., Belov G., Rodionov L. (2015). Visualisation research of the flow processes in the outlet chamber-outlet bridge-inlet chamber zone of the gear pumps. Arch. Civil Mech. Eng..

[33] Banaś M., Antoniak P., Marciniak L., Stryczek J. (2018). Visualization of flow phenomena in hydraulic throttle valves of plastics. MATEC Web Conf..

[34] Shen W., Zhang J., Sun Y., Zhang D.j., Jiang J.h. (2016). Effect of cavitation bubble collapse on hydraulic oil temperature. J. Cent. South Univ..

[35] Discetti S., Coletti F. (2018). Volumetric velocimetry for fluid flows. Measur. Sci. Technol..

[36] Wang H., Xu H., Pooneeth V., Gao X.Z. (2018). A novel one-camera-five-mirror three-dimensional imaging method for reconstructing the cavitation bubble cluster in a water hydraulic valve. Appl. Sci..

[37] Vincent J.F.V., Bogatyreva O.A., Bogatyrev N.R., Bowyer A., Pahl A.K. (2006). Biomimetics: Its practice and theory. J. R. Soc. Interface.

[38] Craig S., Harrison D., Cripps A., Knott D. (2008). BioTRIZ suggests radiative cooling of buildings can be done passively by changing the structure of roof insulation to let longwave infrared pass. J. Bionic Eng..

[39] Jie G., Han S.Z. (2002). A study on the extension AHP method. Syst. Eng..

[40] Vattam S.S., Helms M.E., Goel A.K. (2008). Compound analogical design: Interaction between problem decomposition and analogical transfer in biologically inspired design. Design Computing and Cognition.

[41] Liu Y., Wang Y., Chen X., Yu H. (2018). A spherical conformal contact model considering frictional and microscopic factors based on fractal theory. Chaos Solitons Fractals.

[42] Dekking E.W., Dekking F.M. (2016). Helge von Koch’s snowflake curve revisited. Am. Math. Mon..

[43] Jinsong Z., Weixu S. (2015). Quasisymmetric rigidity of Sierpinski carpets. Ergodic Theory Dyn. Syst..

[44] Yu B., Li J. (2001). Some fractal characters of porous media. Fractals.

[45] Yu B., Lee L.J., Cao H. (2002). A fractal in-plane permeability model for fabrics. Polym. Compos..

[46] William T., Eize J.S. (2014). PIVlab—Towards user-friendly, affordable and accurate digital particle image velocimetry in matlab. J. Open Res. Softw..

